# LESSONS FROM THE RESIDENT EXPERIENCE WITH THE R3 PROGRAM

**DOI:** 10.1093/geroni/igz038.2852

**Published:** 2019-11-08

**Authors:** Edward A Miller, Pamela Nadash, Elizabeth Simpson, Natalie Shellito, Marc Cohen

**Affiliations:** University of Massachusetts Boston, Boston, Massachusetts, United States

## Abstract

Understanding the resident experience is a critical step to creating a sustainable and replicable model of affordable resident-centered housing with supportive services programs. This study thus draws lessons from focus groups with participants in the R3 program for designing and implementing such initiatives in affordable senior housing. Findings indicate that the R3 program brings value to residents: they benefit from reliable information on health-related issues, as well as emotional support and assistance with accessing appropriate care. By focusing on prevention and ensuring timely access to services, findings suggest how the intervention could promote seniors living independently longer and lower health system costs. Results also suggest ways to improve the effectiveness of housing with services programs, including providing clarity regarding the purpose of the program, its components and staffing, building trust between program staff and residents, addressing concerns about privacy and confidentiality, and implementing a multipronged marketing and promotion strategy.

